# Quality and Consumer Acceptability of Organ-Enhanced Ground Beef Patties

**DOI:** 10.3390/foods15173058

**Published:** 2026-08-29

**Authors:** Savannah L. Douglas, Xenia M. Murillo-Contreras, Rilee A. Bennett, Autumn L. Armaly, Sungeun Cho, Don R. Mulvaney, Soren P. Rodning, Jase J. Ball, Jason T. Sawyer

**Affiliations:** 1 Department of Animal Sciences, Auburn University, Auburn, AL 36849, USA; sld0060@auburn.edu (S.L.D.); rzb0145@auburn.edu (R.A.B.); ala0084@auburn.edu (A.L.A.); mulvadr@auburn.edu (D.R.M.); rodnisp@auburn.edu (S.P.R.); jjb0127@auburn.edu (J.J.B.); 2 Department of Poultry Sciences, Auburn University, Auburn, AL 36849, USA; xmm0001@auburn.edu (X.M.M.-C.); szc0181@auburn.edu (S.C.)

**Keywords:** beef, beef heart, beef kidney, edible offal, quality, sensory evaluation, variety meats

## Abstract

Edible beef organs are nutrient-dense ingredients that may improve whole-animal utilization while enhancing the nutritional value of ground beef. This study evaluated the effects of organ type and inclusion level on the quality characteristics and consumer acceptability of ground beef patties. Patties were manufactured from three independent batches and formulated as a control (0% organ), 6% beef heart, 12% beef heart, 6% beef kidney, or 12% beef kidney. Nutritional composition, cooking characteristics, instrumental color, texture, and consumer acceptance were evaluated. Organ-containing patties had higher moisture content than the control (*p* = 0.0082), while 12% beef kidney resulted in the greatest cholesterol and sodium concentrations (*p* < 0.0001). Patties containing 12% beef kidney exhibited the lowest cooking loss (*p* = 0.0004) and Allo–Kramer shear force values (*p* < 0.0001), corresponding to the greatest consumer-perceived tenderness (*p* < 0.0001). However, this treatment received the lowest ratings for overall liking, flavor, texture, purchase intent, and willingness to pay (*p* < 0.0001). In contrast, 6% and 12% beef heart retained consumer acceptability comparable to the control while reducing formulation cost. These findings identify beef heart as a promising ingredient for increasing edible organ utilization in ground beef without compromising consumer acceptance.

## 1. Introduction

The ingredient composition of ground meat products can substantially influence product functionality, eating quality and visual appearance [1,2]. Furthermore, altering meat structure through grinding and forming can impact characteristics such as moisture content, pigment concentration, cooking yield, texture and color stability during retail display and cooking [1,3]. Fresh ground beef purchasing decisions are strongly influenced by visual appearance, particularly color. Consumer acceptance of meat products after purchase in a retail setting is largely determined by palatability attributes such as tenderness, juiciness and flavor [4,5]. Formulation changes in meat products can alter tenderness, juiciness and color characteristics, which may directly influence consumer acceptance [4,5]. Consequently, identifying ingredients that improve nutritional value and product utilization while maintaining desirable quality characteristics remains an important area of interest in meat product development [6].

Edible beef organs represent nutrient-dense ingredients containing elevated concentrations of iron, selenium, zinc, vitamin A, and B-complex vitamins compared with skeletal muscle tissue [7,8,9]. Organ meats have been identified as concentrated sources of highly bioavailable micronutrients capable of contributing to dietary needs [8,10]. Recent evaluations of edible offal products report that organs, such as beef liver, heart and kidney, contain higher concentrations of micronutrients compared to traditional beef cuts while remaining suitable for human consumption [7,9]. Despite these nutritional advantages, edible organs remain underutilized due to concerns associated with flavor, texture, appearance and consumer unfamiliarity [11,12].

In addition to their nutrient density, edible organs have gained renewed interest within the emerging “Food is Medicine” framework, which emphasizes the role of whole foods in disease prevention and health promotion through the consumption of bioavailable nutrients rather than isolated dietary supplements [13,14,15]. Organ meats provide concentrated amounts of protein, iron, zinc, selenium, vitamin A, vitamin B12 and other essential micronutrients that support metabolic function and contribute to overall dietary quality [13,14]. Recent evidence demonstrates that increased organ meat consumption is associated with a lower prevalence of nonalcoholic steatohepatitis, supporting theories that moderate consumption of organ meats may provide benefits beyond nutrition [14]. These human health findings highlight the potential for organ meats to serve not only as a sustainable protein source but also as a functional ingredient aligning with the “Food is Medicine” strategy [14,15].

In addition, the increasing utilization of edible organ meats has been recognized as an important strategy for improving sustainability and economic efficiency within meat production systems [13]. Previous literature has identified opportunities to improve whole-animal utilization through the incorporation of organ tissues into human food products rather than diverting them toward lower-valued applications such as rendering or pet food production [13]. Additionally, nutrient density assessments of animal-derived foods have emphasized the importance of maximizing nutrient yield from existing livestock systems to support future food sustainability goals [6]. Therefore, using edible organs in commonly consumed products provides an opportunity to improve carcass utilization while increasing the nutrient density of fresh beef products [13].

Ground beef represents a practical platform for organ incorporation, as grinding reduces structural differences among tissues and allows ingredient blending within a familiar product [16]. Previous research demonstrates that formulation changes in ground beef systems can alter cooking yield, texture development and color characteristics depending on ingredient composition and moisture retention properties [2,17]. Furthermore, changes in protein functionality and water-binding capacity during thermal processing can substantially influence tenderness and cooking performance in ground meat products [1,3]. Organ tissues differ in structural and biochemical composition, which may influence product quality depending on organ type and inclusion level [18]. Differences in connective tissue and collagen characteristics can influence the textural properties of meat and its response to cooking [19]. Additionally, beef heart and kidney differ in total and heme iron content, with reported differences in the distribution of heme iron between bovine heart and kidney [9]. These compositional differences may contribute to organ-specific effects on texture, cooking characteristics, and instrumental color when these organs are incorporated into ground beef products.

Previous research demonstrates the practicality of incorporating beef heart into ground beef patties [16]. Additionally, a recently published study compared beef heart, liver and kidney at a 15% inclusion rate and identified heart and kidney as contenders for further formulation optimization [20]. However, optimal inclusion rates of these organs while maintaining desirable quality and consumer acceptance remain unclear. Understanding formulation-dependent changes in product functionality is important for identifying formulation thresholds capable of improving organ utilization while maintaining desirable product quality [13,16]. As such, the objective of this study was to compare the effects of beef heart and beef kidney at two levels (6% and 12%) to identify formulations best suited for improving nutrient density while maintaining product quality and consumer acceptability.

## 2. Materials and Methods

### 2.1. Raw Materials, Processing and Packaging

Commercial beef trimmings designated as 80% lean and 20% fat per supplier specification (190 kg; USDA Institutional Meat Purchasing Specifications #138), beef heart (9 kg; USDA Institutional Meat Purchasing Specifications #1723), and beef kidney (9 kg; USDA Institutional Meat Purchasing Specifications #1728) were sourced from a commercial meat supplier (Capital Farms, Wickenburg, AZ, USA; Quincey Cattle Company, Chiefland, FL, USA) and transported under commercial refrigerated conditions to the Lambert-Powell Meats Laboratory (Auburn, AL, USA). All raw materials were obtained from USDA-inspected commercial establishments and were subjected to standardized handling, storage, and processing procedures upon arrival at the laboratory.

Raw materials were randomly allocated to one of three batches for formulation. Three 12.67 kg batches were manufactured for each treatment. Each batch was divided into 5 treatments: control containing no organ (0%), 6% beef heart, 6% beef kidney, 12% beef heart, or 12% beef kidney. Beef trimmings and organs were coarse ground individually through a 9.525 mm plate (SPECO 400, Schiller Park, IL, USA) using a commercial grinder (Model AFMG-48, Biro Manufacturing Company, Marblehead, OH, USA). Following coarse grinding, treatments were mixed for 3 min and subsequently ground through a 3.18 mm plate (SPECO 400, Schiller Park, IL, USA) fitted with a bone eliminator attachment (SPECO 400, Schiller Park, IL, USA). No additional ingredients, including salt, spices, preservatives, binders, or non-meat proteins, were added to any formulation. Ground beef mixtures were portioned into 151.1 g patties (*n* = 76 patties/batch/treatment) using an automated patty forming system (Super 54, Hollymatic Corporation, Countryside, IL, USA).

Formed patties were crust-frozen at −25 °C (Model LEH0630, Larkin, Stone Mountain, GA, USA) for 30 min prior to packaging. Individual patties were packaged using a thermoforming packaging system (Optimus OL0924, Variovac, Zarrentin, Germany) with a forming film oxygen transmission rate of 0.4 cc/m^2^/24 h, a vapor transmission rate of 3.3 g/mm^2^, a non-forming film oxygen transmission rate of 1.0 cc/m^2^/24 h, and a vapor transmission rate of 3.5 g/mm^2^ (WINPAK Ltd., Winnipeg, MB, Canada). Packaged patties were subsequently stored under dark frozen conditions at −22.7 °C (Arctic Air AF49, Broich Enterprises, Minneapolis, MN, USA) until completion of laboratory analyses (7 d).

### 2.2. Nutritional Analysis

Patties were analyzed for nutritional composition by a commercial laboratory (Food Safety Net Services, San Antonio, TX, USA). Three patties from each batch were combined prior to laboratory analysis, resulting in one composite sample per batch per treatment. Sampling was completed after final treatment formulation. Composite samples from each treatment were vacuum packaged and transported under insulated refrigerated conditions for laboratory analysis. Samples were analyzed in triplicate using AOAC Official Methods procedures for moisture content, crude protein, total fat and fatty acid composition, ash and cholesterol concentration [21]. Mineral concentrations, including calcium, iron, potassium, and sodium, were quantified according to corresponding AOAC methods [21]. Total caloric values were calculated from analytical composition values provided by the commercial laboratory.

### 2.3. Cooking Characteristics

Prior to cooking, patties (*n* = 15/batch/treatment) were thawed and held under refrigerated conditions (2.0 ± 1.5 °C) until cooking, with holding times consistent across all treatments and batches. After thawing, patties were removed from packaging, blotted dry with paper towels, and weighed using an analytical balance (Model MA2002P, Mettler-Toledo, Leicester, UK) to determine raw weight.

Patties were cooked in a pre-heated commercial convection oven (Vulcan, Baltimore, MD, USA) set to 176.7 °C until reaching an internal endpoint temperature of 71.1 °C. Internal temperature was monitored using a multichannel temperature data logger (TCTemp X-Series, LogMaster, ThermoWorks, American Fork, UT, USA), with temperature measurements collected at 30 s intervals throughout cooking. Cooking time was defined as the total duration required for samples to achieve the target internal temperature. Following cooking, patties were cooled to room temperature (21.1 °C) and reweighed to determine cooked weight. Cooking loss percentage was calculated as [(raw weight − cooked weight) ÷ raw weight] × 100 [19].

### 2.4. Instrumental Color Evaluation

For raw surface color determination, patties (*n* = 15/batch/treatment) were removed from packaging and allowed to bloom at room temperature with oxygen exposure for 20 min prior to measurement. Cooked internal color measurements were obtained from patties prepared using the previously described cooking procedures. After cooling to room temperature, patties were horizontally sectioned through their geometric center for internal color evaluation.

Color measurements were collected using a HunterLab MiniScan EZ colorimeter (Model 45/0 LAV, Hunter Associates Laboratory Inc., Reston, WV, USA). Instrument calibration was completed prior to data collection using standardized black and white calibration tiles according to manufacturer guidelines. Instrumental color measurements were recorded using illuminant A, a 31.8 mm aperture, and a 10° observer angle. Three readings were collected per patty and averaged for statistical analysis. Measurements included lightness (L*), redness (a*), and yellowness (b*). Hue angle values were calculated as tan^−1^ (b* ÷ a*) to estimate the color shift from red toward yellow, while chroma (C*) values were calculated as √a^2^ + b^2^ to estimate color vividness and saturation. Reflectance values from 400 to 700 nm were additionally used to calculate a red-to-brown ratio (630 nm ÷ 580 nm) to evaluate internal cooked color development [22].

### 2.5. Texture Analysis

Instrumental tenderness was evaluated using a 5-blade Allo–Kramer shear force attachment connected to a texture analyzer (Model TA.XT.Plus 100C, Texture Technologies Corp., New York, NY, USA). Texture profile analysis (TPA) was performed using the same texture analyzer system. Cooked patties (*n* = 15/batch/treatment) were cooled to room temperature at 23.3 °C and sectioned into 6 × 9 cm^2^ portions prior to analysis. Samples were sheared once using a 490.5 N load cell and a crosshead speed of 3 mm/s. Maximum peak force generated during shearing was recorded and reported as Allo–Kramer shear force (N).

Three 1 × 1 cm^2^ sections were removed from each cooked patty and subjected to a two-cycle compression test using a 490.5 N load cell for the completion of texture profile analysis. Samples were compressed to 50% of their original height using a 50 mm cylindrical probe (TA-25A) at a crosshead speed of 6.0 cm/min. Texture attributes were calculated according to previously established procedures and included hardness, cohesiveness, springiness, chewiness, and resilience. Hardness was defined as the force required for sample compression, cohesiveness as the degree of structural integrity prior to fracture, springiness as the ability of the sample to recover after deformation, and chewiness as the energy required for mastication prior to swallowing [23].

### 2.6. Consumer Sensory Panel

Consumer panelists 18 years of age or older who reported consuming ground beef at least two to three times per month were recruited to participate in the study (*n* = 81; Table 1). Study procedures were reviewed and approved as exempt by the Auburn University Institutional Review Board (Exempt Protocol STUDY00001132). Consumer sensory evaluation was conducted across three consecutive testing days to allow presentation of samples from each batch within each treatment. During all testing sessions, panelists evaluated five samples representing one batch from each treatment, resulting in a total of 15 samples evaluated across the study period.

A sample serving order was generated using RedJade sensory software (version 6.1, Pleasant Hill, CA, USA) to ensure randomized serving sequences. Samples were identified using randomly assigned three-digit blinding codes prior to evaluation. Ground beef patties (*n* = 25/batch/treatment) were cooked to an internal endpoint temperature of 71.1 °C and sectioned into triangular portions according to American Meat Science Association sensory evaluation guidelines [19]. Prepared samples were served individually to panelists in randomized order within private sensory booths under red color-masked lighting conditions to minimize visual color bias during evaluation.

Panelists were instructed to cleanse their palate between samples using room-temperature water and unsalted saltine crackers (Premium Unsalted Tops, Nabisco, East Hanover, NJ, USA). Consumers evaluated overall liking, aroma, flavor, and texture using a 9-point hedonic scale (1 = dislike extremely; 9 = like extremely). Perceived intensity of juiciness, tenderness, and beef flavor was additionally evaluated using 9-point intensity scales, where juiciness ranged from 1 = extremely dry to 9 = extremely juicy, tenderness ranged from 1 = extremely tough to 9 = extremely tender, and beef flavor intensity ranged from 1 = none to 9 = extremely intense. Following evaluation, panelists selected descriptive sensory characteristics applicable to each sample, including beefy, liver-like, metallic, juicy, dry, tender, rancid, gamey or fatty. Lastly, consumers were asked to indicate purchase intent (1 = definitely would not purchase; 9 = definitely would purchase) and willingness to pay for the selected sample on a scale from 1 = would not buy this product for any price to 6 = $22.05 or more per kg.

### 2.7. Formulation Cost Comparison

An estimated formulation cost was calculated for each treatment to evaluate the potential economic impact of beef heart and beef kidney usage in ground beef patties. Ingredient costs were calculated using average market prices for 80:20 beef trimmings, beef heart and beef kidney at the time of product formulation. Purchase prices were $13.23/kg for 80:20 beef trimmings, $8.27/kg for beef heart, and $4.96/kg for beef kidney (Capital Farms, Wickenburg, AZ, USA). Cost per kilogram was calculated based on the proportion of each ingredient included in the treatment formulation. Cost reduction was calculated by subtracting the estimated treatment cost from the control formulation cost, and percent reduction was calculated as [(control cost − treatment cost) ÷ control cost] × 100. Cost estimates were based on ingredient substitution only and did not include additional expenses associated with labor, processing, packaging, storage, distribution or retail markup.

### 2.8. Statistical Analysis

Data was analyzed using the GLIMMIX procedure of SAS (version 9.4; SAS Institute Inc., Cary, NC, USA). Physicochemical and instrumental measurements (cook loss, cook time, raw and cooked color, Allo–Kramer shear force, and texture profile analysis) were analyzed using treatment as a fixed effect and batch as a random blocking factor. Patties produced within each batch served as experimental units for each analysis. Consumer sensory ratings were analyzed with treatment as a fixed effect, while batch and panelist were treated as random effects. For descriptive sensory attribute selection frequencies, each sensory descriptor was analyzed as a binary response (selected or not selected). Data was analyzed using the GLIMMIX procedure with a binomial distribution and logit link function, with treatment included as a fixed effect and panelist as a random effect. Means were separated using pairwise comparisons with the PDIFF option, and significance was determined at *p* ≤ 0.05.

## 3. Results and Discussion

### 3.1. Nutritional Composition

With increasing consumer interest in nutrient-dense foods, nutritional composition is an important consideration when evaluating novel meat products. The nutritional composition of ground beef patties formulated with beef heart and kidney is presented in Table 2. Treatment affected moisture (*p* = 0.0082), caloric density (*p* = 0.0301), cholesterol (*p* = 0.0002), potassium (*p* = 0.0158) and sodium (*p* < 0.0001), whereas protein (*p* = 0.5596), total fat (*p* = 0.1266), ash (*p* = 0.3302), iron (*p* = 0.2909), and calcium (*p* = 0.8879) were not affected by treatment. Caloric density was lower in all organ-containing treatments than in the control (*p* = 0.0301). Cholesterol concentration increased with organ usage (*p* = 0.0002), with kidney 12% patties exhibiting the greatest cholesterol concentration and the control (0%) exhibiting the lowest. Potassium (*p* = 0.0158) concentration was greatest in kidney 12% patties and lowest in kidney 6% patties, while sodium (*p* < 0.0001) concentration was greatest in kidney 12% patties compared to all other treatments.

All treatments containing organs exhibited higher moisture percentages relative to control patties (*p* = 0.0082). Increased moisture content likely reflects the higher intracellular water content and reduced lipid concentration of organ tissues compared with adipose tissue traditionally present within 80:20 ground beef formulations [7,8]. Similar increases in moisture content have previously been reported in reformulated meat products containing alternative lean ingredients where substitution altered water retention and overall product composition [17,20].

Despite differences in moisture, protein concentration did not differ among treatments (*p* = 0.5596), indicating that beef heart and kidney contributed protein concentrations comparable to muscle tissue [7,10]. Likewise, total fat content did not differ among treatments (*p* = 0.1266). However, caloric density was lower in all organ-containing treatments compared to the control. This reduction in caloric density likely reflects the numerical decrease in fat content coupled with increased moisture, effectively diluting the overall energy density of the product. The current study results suggest that the use of organ meats may improve nutrient density while reducing caloric concentration.

Cholesterol concentration increased with organ inclusion (*p* = 0.0002). Patties containing 12% kidney exhibited the greatest cholesterol concentration, whereas the control exhibited the lowest. Heart and kidney treatments exhibited intermediate cholesterol concentrations that generally increased with organ supplementation level. Organ tissues contain higher concentrations of phospholipids and sterol compounds than skeletal muscle tissue, which likely contributed to the observed differences [7,9]. Potassium (*p* < 0.0001) and sodium (*p* < 0.0001) concentrations were influenced by treatment, with kidney 12% patties exhibiting the greatest concentrations of both minerals. Potassium is an important dietary mineral, and intake is commonly below recommended levels in the U.S. population [24]. Increased electrolyte concentrations likely reflect the physiological role of renal tissues in filtration and nutrient transport [12]. Utilizing organ meats may provide an opportunity to enhance micronutrient density while maintaining protein concentration. Overall, the incorporation of beef heart and kidney altered the nutritional composition of ground beef patties while maintaining protein concentration and reducing caloric density.

### 3.2. Cooking Characteristics

Modification of product formulations can lead to increased cooking loss, which can result in decreased consumer liking and financial impact for the food service industry [2]. Cooking characteristics of ground beef patties are presented in Table 3. Treatment influenced cooking loss percentage (*p* = 0.0004), whereas cooking time was not affected (*p* = 0.1396). Heart 12% patties exhibited the greatest numerical cooking loss and differed from kidney 12% patties, which exhibited the lowest cooking loss. Reduced cooking loss observed in patties containing kidney likely resulted from greater moisture retention associated with the less organized structure and higher intracellular water content of renal tissue [2,3]. Increased moisture retention may have limited fluid migration during cooking, causing a reduction in total loss [1]. Similar results have been reported in reformulated ground meat products containing alternative protein ingredients [17,20].

In contrast, patties containing heart exhibited increased cooking loss despite having similar moisture concentrations to patties containing kidney. Cardiac muscle contains densely organized contractile fibers, which may promote greater moisture expulsion during protein denaturation compared to kidney tissues [18]. Although heart 12% exhibited the greatest numerical cooking loss, sensory texture and overall liking ratings remained comparable to the control, suggesting that the observed differences in cooking loss were insufficient to negatively affect consumer acceptance.

Despite differences in cooking loss, treatment did not affect cooking time. Similar cooking times among treatments suggest that substitution of beef heart or kidney at either inclusion level did not alter heat transfer or the rate at which patties reached the target internal temperature. These findings indicate that organ incorporation influenced moisture retention during cooking without affecting overall cooking performance.

### 3.3. Instrumental Color Analysis

Raw surface color is one of the most influential quality attributes affecting consumer purchasing decisions for fresh beef [4,25]. Surface color is regulated by myoglobin concentration and oxygen exposure [2,25]. Instrumental raw surface colors are presented in Table 4. The main effect of treatment indicated differences among treatments for raw surface color, including lightness (*p* < 0.0001), redness (*p* < 0.0001), yellowness (*p* = 0.0008), hue angle (*p* = 0.0019) and chroma values (*p* < 0.0001). Kidney 6% patties exhibited the greatest redness and chroma values, whereas the control exhibited the lowest values. Kidney 12% patties exhibited redness values similar to both heart treatments but greater than the control. Lightness decreased, with kidney 12% exhibiting the lowest L* values among all the treatments. Lightness values decreased with increasing kidney inclusion, suggesting that an increase in pigment concentration reduced surface reflectance characteristics [25,26]. Decreases in lightness have previously been reported in beef products containing elevated myoglobin concentrations and darker muscle tissues [25,26]. Patties containing kidney exhibited greater surface redness than the control, with kidney 6% exhibiting the greatest redness. Patties containing heart had intermediate redness values similar to kidney 12% samples. Increasing formulation levels from 6% to 12% did not further intensify redness within either organ type. These findings suggest that the incorporation of organ tissue rather than the formulation level itself was primarily responsible for the increase in red color intensity. Increased redness observed in patties containing kidney is likely associated with elevated concentrations of heme pigments and iron-containing compounds commonly present in visceral tissues [9]. Similar relationships between pigment concentration and increased red color intensity have been reported in beef products containing organ tissues and iron-rich ingredients [15]. Additionally, increased chroma values support increased color saturation and visual intensity following organ supplementation. Total color difference (ΔE) provides a measure of differences in L*, a*, and b* values relative to a reference and has been used to characterize overall color differences in ground beef products [22]. Despite differences among individual color parameters, ΔE did not differ among treatments (*p* = 0.3208). This indicates that organ inclusion did not significantly alter overall raw surface color relative to the control. Similarly, a ground beef study evaluating the addition of dried beans, citrus fiber, potato peel and potato extract reported that no difference in color change was detected compared to the control [27].

Cooked color values are an important factor to assess, as persistent pinking in ground beef products can lead consumers to perceive patties as undercooked during consumption [28,29]. Cooked internal color values are presented in Table 5. Treatment influenced lightness (*p* < 0.0001), redness (*p* = 0.0003), yellowness (*p* < 0.0001), hue angle (*p* < 0.0001) and chroma values (*p* < 0.0001). Despite significant differences in individual color parameters, total cooked color difference (ΔE) did not differ among treatments (*p* = 0.7511), indicating that organ inclusion did not significantly alter overall cooked color relative to the control. Patties containing 12% kidney inclusion exhibited the greatest cooked redness and chroma values, whereas control samples and samples containing 12% heart exhibited the lowest values. Patties containing kidney exhibited lower hue angle values compared to the control, indicating greater retention of red color following cooking. Persistent redness observed in kidney patties may be associated with tissue-specific differences in heme-containing compounds [9]. Differences in heme iron distribution between bovine heart and kidney, with a greater proportion of total iron present as heme iron in kidney, have previously been reported [9]. It is plausible that the persistent redness observed in kidney 12% patties was due to the elevated concentration of heat-stable heme pigments and altered myoglobin denaturation during cooking [1,25]. Increased moisture retention within kidney-containing treatments may have limited denaturation during cooking, resulting in preserved red color intensity after cooking [3]. Persistent redness has previously been reported in beef products formulated with increased myoglobin concentrations and iron-rich ingredients [28,30]. Control patties exhibited greater hue angle values relative to treatments containing kidney, indicating a greater shift away from red color characteristics. While kidney increased redness, patties containing heart exhibited similar cooked color characteristics to control patties, which may reduce concerns associated with consumer perceptions of doneness. Further research should be conducted to evaluate the implications of persistent pinking associated with the use of organ meats and the potential influence these color changes may have on consumer perception of doneness, product safety and purchasing intent under traditional retail and consumption conditions [29].

### 3.4. Texture Analysis

Objective texture characteristics of ground beef patties formulated with beef heart and kidney inclusion are presented in Table 6. Treatment influenced Allo–Kramer shear force (*p* < 0.0001), hardness (*p* < 0.0001), springiness (*p* = 0.0006), cohesiveness (*p* < 0.0001), chewiness (*p* < 0.0001) and resilience (*p* < 0.0001). Kidney 12% patties exhibited the lowest Allo–Kramer shear force (AKSF), hardness, chewiness and resilience values among treatments, whereas heart 6% patties exhibited the greatest AKSF and hardness values. Springiness was lower in both treatments containing kidney than in the control and heart-containing treatments.

Reduced hardness and chewiness values suggest that kidney incorporation produced a softer and less rigid product. Reductions in texture values are likely associated with the less organized structure and increased moisture retention of renal tissue compared to skeletal and cardiac muscle [18]. Greater moisture retention likely softened the product, leading to reduced resistance during compression and mastication [3]. Similar changes in instrumental texture have been reported in reformulated ground meat products containing alternative protein ingredients. A previous study evaluated ground beef patties formulated with 3.5% oat, pea, or rice protein in combination with textured vegetable protein and soy protein concentrate and reported greater Allo–Kramer shear force values compared with the control, indicating reduced tenderness [17]. Moreover, reformulation of meat products with non-meat ingredients can alter texture through changes in water retention, protein interactions, and product structure [20].

In contrast, patties containing heart generally exhibited texture values similar to or greater than control patties. Heart 6% patties exhibited the greatest hardness and shear force values among all treatments. Cardiac muscle contains highly organized contractile fibers adapted for continual contraction, which likely contributed to greater structural integrity and increased firmness following cooking [18]. Previous studies evaluating muscle structure and connective tissue organization reported increased structural integrity and shear force values in products containing greater connective tissue [30]. Despite increased instrumental firmness, patties containing heart matched sensory texture ratings comparable to those of control patties, suggesting these differences were not sufficient to negatively influence consumer palatability.

### 3.5. Consumer Acceptance

Consumer panelists (*n* = 81) evaluated sensory characteristics of ground beef patties formulated with 6% or 12% inclusion of heart or kidney to determine consumer acceptance of ground beef formulations containing organ meats. Consumer acceptance scores are presented in Table 7. Differences among treatments were observed for overall liking (*p* < 0.0001), flavor (*p* < 0.0001) and texture (*p* < 0.0001), whereas aroma retained similar scores among treatments (*p* = 0.5689). Patties containing heart and 6% kidney had consistent overall liking, flavor, and texture ratings comparable to the control’s, whereas kidney 12% received lower ratings than all other treatments.

Reduced liking associated with kidney 12% patties is likely due to an increased liver-like and metallic flavor associated with kidney tissue. These flavor attributes have previously been associated with oxidative flavor compounds and elevated iron concentrations in meat products [31]. Similar declines in consumer acceptance have been reported when organ levels exceed concentrations capable of masking characteristic offal flavors within processed meat products; however, sensory effects are likely dependent on the specific organ type and inclusion rate [11]. In contrast, the incorporation of beef heart at either inclusion level resulted in consumer acceptance comparable to the control, suggesting that beef heart may provide an opportunity to improve edible organ utilization without negatively affecting eating quality.

Consumer sensory intensity ratings are presented in Table 8. Treatment influenced juiciness (*p* = 0.0105), tenderness (*p* < 0.0001), and beef flavor intensity (*p* < 0.0001). Heart 6% patties received lower juiciness ratings than all other treatments, whereas kidney 12% patties received the greatest tenderness ratings. Beef flavor intensity increased with kidney inclusion, with kidney 12% receiving greater ratings than the control and both treatments containing heart.

Greater tenderness ratings in kidney 12% patties correspond with reduced cooking loss and lower instrumental shear force values, suggesting increased moisture retention contributed to improved palatability [2,3]. Similar relationships between increased moisture retention and tenderness have previously been reported in reformulated meat systems and organ-containing products [9,10]. Despite greater cooking loss, patties containing beef heart were similar to the control in tenderness and beef flavor intensity ratings. These results indicate that instrumental differences observed in cooking characteristics were not sufficient to reduce consumer perception of eating quality.

Consumer selection frequencies for descriptive attributes are presented in Table 9. Treatment differences were observed for liver-like (*p* < 0.0001), metallic (*p* = 0.0184), fatty (*p* = 0.0163), and dry (*p* < 0.0001) descriptors. Frequencies of beefy, juicy, tender, gamey and rancid descriptors did not differ among treatments. Kidney 12% patties were most frequently described as liver-like and metallic, whereas patties containing heart exhibited descriptor frequencies similar to the control. Conversely, dry was selected less frequently for patties containing kidney, particularly kidney 12%, than for the control and heart 6%. Increased metallic and liver-like flavor perception likely resulted from elevated iron concentrations and oxidative flavor compounds naturally associated with kidney tissue [9]. Increases in metallic and organ-associated flavor notes have been reported in studies evaluating organ-containing meat products and oxidation-derived flavor compounds [11].

Consumer purchase intent and willingness to pay are presented in Table 10. Treatment influenced both purchase intent (*p* < 0.0001) and willingness to pay (*p* < 0.0001). Patties containing heart received similar purchase intent and willingness-to-pay ratings to the control. Kidney 12% received lower ratings than all other treatments. These results are consistent with overall liking responses and indicate that consumers were willing to purchase and pay comparable prices for products containing beef heart but were less likely to purchase products containing kidney. Similar relationships between overall liking, purchase intent, and willingness to pay have been reported in consumer studies evaluating beef quality perception and purchasing behavior [32].

### 3.6. Formulation Cost Comparison

Estimated formulation costs for ground beef patties containing beef heart and kidney are presented in Table 11. Formulation cost decreased as organ inclusion increased, regardless of organ type. As expected, control patties exhibited the greatest estimated cost ($13.23/kg), whereas kidney 12% patties exhibited the lowest estimated formulation cost ($12.24/kg), which represents a 7.48% reduction in cost relative to the control. Heart 12% and kidney 6% formulations reduced estimated formulation costs by 4.53% and 3.73%, while heart 6% reduced formulation costs by 2.19%. Cost reductions observed in organ-containing treatments were expected, as edible organs are generally lower-value carcass components compared with skeletal muscle cuts despite possessing substantial nutritional value [12]. Replacing a portion of traditional beef trim with heart or kidney reduced overall ingredient costs while maintaining product functionality. Similar opportunities for value addition through the incorporation of edible by-products have been identified as an important strategy for improving whole-animal utilization and increasing sustainability within meat production systems [12].

Although kidney 12% resulted in the greatest reduction in formulation cost, these savings were accompanied by reduced consumer acceptance and willingness to pay. Conversely, heart 12% reduced formulation costs by 4.53% while maintaining overall liking, purchase intent, and willingness to pay comparable to those of the control. Therefore, beef heart appears to provide the most favorable balance among economic value, product quality, and consumer acceptance.

It is important to note that although cost reductions at the 6% inclusion level were relatively modest on a per-kilogram basis, these differences may become more economically relevant in large-scale commercial production where incremental savings can accumulate across greater production volumes. However, the cost estimates presented represent formulation costs only and do not account for processing, labor, packaging, distribution, or other factors that would influence commercial profitability.

## 4. Conclusions

The addition of beef heart and beef kidney to ground beef patties altered nutritional composition, physicochemical characteristics and consumer sensory responses. Organ incorporation increased moisture content and reduced caloric density while maintaining protein concentrations comparable to those of control patties. Patties containing kidney exhibited reduced cooking loss, lower instrumental shear force values, and increased redness in both raw and cooked products. This suggests improvements in moisture retention and altered pigmentation associated with organ incorporation. Patties containing 12% kidney had greater cholesterol concentrations and differed in instrumental color while receiving lower consumer acceptance scores. In contrast, beef heart had sensory ratings for overall liking, flavor, aroma, and texture that were comparable to those of control patties while providing nutritional benefits associated with organ incorporation. However, changes in sodium and cholesterol concentrations should be considered when evaluating the overall nutritional implications of organ incorporation. Among the organs evaluated, beef heart represented the most successful formulation strategy by maintaining consumer acceptance while improving organ utilization and reducing formulation cost. These findings suggest beef heart could be incorporated into value-added ground beef products for retail and food service applications, allowing processors to improve carcass utilization while offering a nutrient-dense product that maintains consumer acceptability. Further research should evaluate processing feasibility and economic benefits under commercial production conditions. Oxidative stability during frozen or refrigerated storage was not evaluated in the current study. Given the high heme iron content and other pro-oxidant components of organ tissue, incorporation may increase susceptibility to lipid oxidation during extended storage. As the present study focused on formulation optimization, subsequent shelf-life studies should assess oxidative stability and storage quality to determine the commercial viability of organ-enhanced ground beef products.

## Figures and Tables

**Table 1:** Consumer sensory panel demographics.

	Respondents	Percentage
Sex		
Male	33	40.74%
Female	48	59.26%
Age		
18 to 20 years	3	3.70%
21 to 29 years	56	69.14%
30 to 39 years	12	14.81%
40 to 49 years	6	7.41%
50 to 59 years	1	1.23%
60 to 69 years	2	2.47%
70 to 79 years	1	1.24%
Ethnicity		
White or Caucasian	47	58.02%
Hispanic or Latino	17	20.99%
Black or African American	5	6.17%
Native American or American Indian	0	0.00%
Asian or Pacific Islander	12	14.81%
Income		
Less than USD 30,000	58	71.60%
USD 30,000 to USD 49,999	11	13.58%
USD 50,000 to USD 79,999	6	7.42%
Greater than USD 80,000	6	7.42%
Purchases Ground Beef		
Once a week or more often	37	45.70%
Once every 2 or 3 weeks	29	35.80%
Once a month	12	14.81%
Once every 2 or 3 months	1	1.23%
Once every 4 to 6 months	0	0.00%
Once or twice a year	1	1.23%
Less than once a year	1	1.23%
Consumes Ground Beef		
Once a day or more	3	3.70%
More than 3 times per week	19	23.46%
2 to 3 times per week	32	39.51%
Once a week	18	22.22%
2 to 3 times per month	9	11.11%
Typical Price Paid Per Pound of Ground Beef		
USD 6.00 to 6.99	23	28.40%
USD 7.00 to 7.99	27	33.33%
USD 8.00 to 8.99	23	28.40%
USD 9.00 to 9.99	5	6.17%
USD 10.00 or more	3	3.70%

**Table 2:** Nutrient density of ground beef patties with or without heart and kidney inclusion.

	Treatments						
Nutrients	Control	Heart 6%	Heart 12%	Kidney 6%	Kidney 12%	SEM *	*p*-Value
Moisture (%)	62.13 ^b^	64.85 ^a^	64.88 ^a^	64.45 ^a^	65.24 ^a^	0.729	0.0082
Protein (%)	18.75	18.14	18.95	18.53	18.42	0.421	0.5596
Total Fat (g/100 g)	17.96	15.09	15.92	15.05	14.92	0.942	0.1266
Ash (%)	0.87	0.86	0.99	1.06	1.10	0.095	0.3302
Calories (kcal/100 g)	238.46 ^a^	212.60 ^b^	219.09 ^b^	212.90 ^b^	208.80 ^b^	7.015	0.0301
Cholesterol (mg/100 g)	69.03 ^d^	74.73 ^cd^	81.01 ^bc^	86.70 ^b^	99.56 ^a^	2.863	0.0002
Iron (mg/100 g)	2.11	2.19	2.98	2.36	2.69	0.304	0.2909
Potassium mg/100 g	289.83 ^bc^	299.81 ^ab^	299.33 ^ab^	274.85 ^c^	307.78 ^a^	6.089	0.0158
Sodium (mg/100 g)	53.35 ^bc^	51.31 ^c^	50.93 ^c^	54.90 ^b^	69.09 ^a^	1.147	<0.0001
Calcium (mg/100 g)	4.29	3.63	3.72	4.04	4.03	0.513	0.8879

^a–d^ Mean values within a row lacking common superscripts differ (*p* < 0.05). * SEM, standard error of the mean. Control = ground beef patty with no organ inclusion; heart 6% = ground beef patty formulated with 6% beef heart; heart 12% = ground beef patty formulated with 12% beef heart; kidney 6% = ground beef patty formulated with 6% beef kidney; kidney 12% = ground beef patty formulated with 12% beef kidney.

**Table 3:** Change in cook loss and cook time of ground beef patties.

	Treatments						
	Control	Heart 6%	Heart 12%	Kidney 6%	Kidney 12%	SEM *	*p*-Value
Cook Loss (%) ^1^	30.61 ^ab^	31.09 ^a^	32.39 ^a^	28.58 ^bc^	26.40 ^c^	0.951	0.0004
Cook Time (s) ^2^	1263.69	904.25	929.89	1264.75	1080.03	124.466	0.1396

^a–c^ Mean values within a row lacking common superscripts differ (*p* < 0.05). * SEM, standard error of the mean. ^1^ Cooking loss percentage was calculated as [(raw weight − cooked weight) ÷ raw weight] × 100. ^2^ Cooking time was defined as the total duration required for samples to achieve the target internal temperature (71.1°C). Control = ground beef patty with no organ inclusion; heart 6% = ground beef patty formulated with 6% beef heart; heart 12% = ground beef patty formulated with 12% beef heart; kidney 6% = ground beef patty formulated with 6% beef kidney; kidney 12% = ground beef patty formulated with 12% beef kidney.

**Table 4:** Fresh surface color of control and organ-enhanced ground beef patties.

	Treatments						
	Control	Heart 6%	Heart 12%	Kidney 6%	Kidney 12%	SEM *	*p*-Value
Lightness (L*) ^1^	45.18 ^bc^	45.39 ^b^	45.78 ^a^	44.97 ^c^	44.33 ^d^	0.189	<0.0001
Redness (a*) ^2^	25.39 ^c^	26.41 ^b^	26.40 ^b^	26.96 ^a^	26.61 ^ab^	0.169	<0.0001
Yellowness (b*) ^3^	19.41 ^b^	19.90 ^a^	20.08 ^a^	20.14 ^a^	19.95 ^a^	0.139	0.0008
Hue Angle (°) ^4^	37.42 ^a^	36.99 ^b^	37.29 ^a^	36.74 ^b^	36.84 ^b^	0.114	0.0019
Chroma (C*) ^5^	31.96 ^c^	33.07 ^b^	33.13 ^ab^	33.66 ^a^	33.26 ^ab^	0.165	<0.0001
Color Change (ΔE) ^6^	0.00	1.53	1.50	1.89	1.76	0.224	0.3208

^a–d^ Mean values within a row lacking common superscripts differ (*p* < 0.05). * SEM, standard error of the mean. ^1^ Lightness (L*)—values are a measure of darkness to lightness (a larger value indicates a lighter color); ^2^ redness (a*)—values are a measure of redness (a larger value indicates a redder color); and ^3^ yellowness (b*)—values are a measure of yellowness (a larger value indicates a more yellow color). ^4^ Hue angle (°) represents the change in color from the true red axis (a larger number indicates a greater shift from red to yellow). ^5^ Chroma (C*) is a measure of total color (a larger number indicates a more vivid color). ^6^ Color difference (ΔE) represents the total color difference of each treatment relative to the control. Control = ground beef patty with no organ inclusion; heart 6% = ground beef patty formulated with 6% beef heart; heart 12% = ground beef patty formulated with 12% beef heart; kidney 6% = ground beef patty formulated with 6% beef kidney; kidney 12% = ground beef patty formulated with 12% beef kidney.

**Table 5:** Instrumental cooked color of ground beef patties with or without beef heart and kidney inclusion.

	Treatments						
	Control	Heart 6%	Heart 12%	Kidney 6%	Kidney 12%	SEM *	*p*-Value
Lightness (L*) ^1^	56.15 ^c^	56.67 ^b^	57.08 ^a^	56.02 ^c^	55.03 ^d^	0.926	<0.0001
Redness (a*) ^2^	17.25 ^c^	19.52 ^b^	16.99 ^c^	20.49 ^b^	21.67 ^a^	0.516	0.0003
Yellowness (b*) ^3^	19.11 ^c^	20.28 ^b^	19.48 ^c^	21.25 ^a^	21.56 ^a^	0.661	<0.0001
Hue Angle (°) ^4^	47.57 ^a^	45.81 ^b^	47.88 ^a^	45.57 ^b^	44.34 ^b^	0.584	<0.0001
Chroma (C*) ^5^	25.36 ^c^	27.86 ^b^	25.41 ^c^	29.14 ^ab^	30.15 ^a^	0.510	<0.0001
Color Change (ΔE) ^6^	0.00	4.53	3.44	4.05	5.21	1.286	0.7511

^a–d^ Mean values within a row lacking common superscripts differ (*p* < 0.05). * SEM, standard error of the mean. ^1^ Lightness (L*)—values are a measure of darkness to lightness (a larger value indicates a lighter color); ^2^ redness (a*)—values are a measure of redness (a larger value indicates a redder color); and ^3^ yellowness (b*)—values are a measure of yellowness (a larger value indicates a more yellow color). ^4^ Hue angle (°) represents the change in color from the true red axis (a larger number indicates a greater shift from red to yellow). ^5^ Chroma (C*) is a measure of total color (a larger number indicates a more vivid color). ^6^ Color difference (ΔE) represents the total color difference of each treatment relative to the control. Control = ground beef patty with no organ inclusion; heart 6% = ground beef patty formulated with 6% beef heart; heart 12% = ground beef patty formulated with 12% beef heart; kidney 6% = ground beef patty formulated with 6% beef kidney; kidney 12% = ground beef patty formulated with 12% beef kidney.

**Table 6:** Instrumental tenderness values of control and organ-enhanced ground beef patties.

	Treatments						
	Control	Heart 6%	Heart 12%	Kidney 6%	Kidney 12%	SEM *	*p*-Value
AKSF (N)	310.64 ^ab^	327.84 ^a^	298.78 ^b^	262.70 ^c^	234.87 ^d^	7.767	<0.0001
Hardness (kg)	2.12 ^b^	2.35 ^a^	2.29 ^ab^	1.88 ^c^	1.63 ^d^	8.193	<0.0001
Springiness ^1^	0.81 ^a^	0.82 ^a^	0.83 ^a^	0.79 ^b^	0.79 ^b^	0.006	0.0006
Cohesiveness ^2^	0.51 ^a^	0.52 ^a^	0.52 ^a^	0.51 ^a^	0.49 ^b^	0.004	<0.0001
Chewiness ^3^	915.15 ^a^	1008.86 ^a^	977.20 ^a^	776.81 ^b^	645.17 ^c^	39.466	<0.0001
Resilience ^4^	0.20 ^a^	0.19 ^ab^	0.19 ^b^	0.19 ^ab^	0.19 ^c^	0.002	<0.0001

^a–d^ Mean values within a row lacking common superscripts differ (*p* < 0.05). * SEM, standard error of the mean; ^1^ springiness—ratio of the time duration of force input during the second compression to that during the first compression or length 2 ÷ length 1; ^2^ cohesiveness—ratio of the positive force area during the second compression to that during the first compression, or area 2 ÷ area 1; ^3^ chewiness—hardness × cohesiveness × springiness; ^4^ resilience—ratio of the time duration of force input during the first compression, or area 5 ÷ area 4. Control = ground beef patty with no organ inclusion; heart 6% = ground beef patty formulated with 6% beef heart; heart 12% = ground beef patty formulated with 12% beef heart; kidney 6% = ground beef patty formulated with 6% beef kidney; kidney 12% = ground beef patty formulated with 12% beef kidney.

**Table 7:** Consumer panelists’ overall sensory ratings for ground beef samples with or without heart and kidney inclusion.

	Treatments						
	Control	Heart 6%	Heart 12%	Kidney 6%	Kidney 12%	SEM *	*p*-Value
Overall Liking	6.30 ^a^	6.21 ^a^	6.34 ^a^	6.10 ^a^	5.63 ^b^	0.140	<0.0001
Overall Aroma	6.49	6.51	6.44	6.51	6.33	0.143	0.5689
Overall Flavor	6.31 ^a^	6.41 ^a^	6.39 ^a^	6.18 ^a^	5.69 ^b^	0.133	<0.0001
Overall Texture	6.27 ^a^	6.21 ^a^	6.21 ^a^	6.14 ^a^	5.55 ^b^	0.223	<0.0001

^a,b^ Mean values within a row lacking common superscripts differ (*p* < 0.05). * SEM, standard error of the mean. Control = ground beef patty with no organ inclusion; heart 6% = ground beef patty formulated with 6% beef heart; heart 12% = ground beef patty formulated with 12% beef heart; kidney 6% = ground beef patty formulated with 6% beef kidney; kidney 12% = ground beef patty formulated with 12% beef kidney.

**Table 8:** Consumer panelists’ intensity ratings of control and organ-enhanced ground beef patties.

	Treatments						
	Control	Heart 6%	Heart 12%	Kidney 6%	Kidney 12%	SEM *	*p*-Value
Juiciness	6.26 ^a^	5.79 ^b^	6.17 ^a^	6.16 ^a^	6.25 ^a^	0.200	0.0105
Tenderness	5.81 ^bc^	5.69 ^c^	6.03 ^b^	6.00 ^b^	6.47 ^a^	0.301	<0.0001
Beef Flavor Intensity	5.92 ^c^	5.94 ^c^	6.14 ^bc^	6.33 ^ab^	6.59 ^a^	0.154	<0.0001

^a–c^ Mean values within a row lacking common superscripts differ (*p* < 0.05). * SEM, standard error of the mean. Control = ground beef patty with no organ inclusion; heart 6% = ground beef patty formulated with 6% beef heart; heart 12% = ground beef patty formulated with 12% beef heart; kidney 6% = ground beef patty formulated with 6% beef kidney; kidney 12% = ground beef patty formulated with 12% beef kidney.

**Table 9:** Consumer selection frequency (%) of sensory descriptors for ground beef samples with or without organ inclusion.

	Treatments						
	Control	Heart 6%	Heart 12%	Kidney 6%	Kidney 12%	SEM *	*p*-Value
Beefy (%)	60.63	67.63	64.45	69.18	58.97	0.227	0.1869
Liver (%)	4.10 ^c^	4.65 ^c^	7.05 ^c^	20.83 ^b^	32.23 ^a^	0.285	<0.0001
Metallic (%)	10.63 ^c^	8.43 ^c^	10.63 ^c^	18.94 ^b^	27.16 ^a^	0.258	<0.0001
Fatty (%)	14.78 ^a^	7.98 ^b^	12.39 ^ab^	12.78 ^ab^	18.15 ^a^	0.246	0.0163
Juicy (%)	40.22	35.88	41.52	42.83	48.95	0.153	0.0781
Dry (%)	33.01 ^a^	35.94 ^a^	28.84 ^ab^	22.61 ^bc^	16.03 ^c^	0.158	<0.0001
Tender (%)	48.97	44.83	41.50	48.05	55.41	0.166	0.2655
Gamey (%)	0.30	1.23	0.30	1.86	2.19	0.849	0.1611
Rancid (%)	2.05	0.85	1.44	2.05	3.34	0.466	0.2530

^a–c^ Mean values within a row lacking common superscripts differ (*p* < 0.05). * SEM, standard error of the mean. Control = ground beef patty with no organ inclusion; heart 6% = ground beef patty formulated with 6% beef heart; heart 12% = ground beef patty formulated with 12% beef heart; kidney 6% = ground beef patty formulated with 6% beef kidney; kidney 12% = ground beef patty formulated with 12% beef kidney.

**Table 10:** Consumer purchase intent and willingness to pay of various ground beef formulations.

	Treatments						
	Control	Heart 6%	Heart 12%	Kidney 6%	Kidney 12%	SEM *	*p*-Value
Purchase Intent	5.68 ^a^	5.61 ^a^	5.72 ^a^	5.49 ^a^	4.80 ^b^	0.186	<0.0001
Willingness to Pay	2.80 ^a^	2.74 ^a^	2.82 ^a^	2.71 ^a^	2.42 ^b^	0.104	<0.0001

^a,b^ Mean values within a row lacking common superscripts differ (*p* < 0.05). * SEM, standard error of the mean. Consumer panelist ratings represent the use of a hedonic scale for purchase intent (1 = definitely would not purchase; 9 = definitely would purchase) and willingness to pay (1 = would not buy this product for any price to 6 = $22.05 or more per kg). Control = ground beef patty with no organ inclusion; heart 6% = ground beef patty formulated with 6% beef heart; heart 12% = ground beef patty formulated with 12% beef heart; kidney 6% = ground beef patty formulated with 6% beef kidney; kidney 12% = ground beef patty formulated with 12% beef kidney.

**Table 11:** Estimated formulation cost of ground beef patties with or without beef kidney and heart inclusion.

Treatments	Cost ($/kg)	Cost Reduction ($/kg)	Percent Reduction (%)
Control	$13.23	$0.00	0%
Heart 6%	$12.94	$0.29	2.19%
Heart 12%	$12.63	$0.60	4.53%
Kidney 6%	$12.74	$0.49	3.73%
Kidney 12%	$12.24	$0.99	7.48%

Values represent estimated formulation cost based on average market prices. Cost estimates are based on ingredient substitution and do not account for processing, labor, or distribution costs. Control = ground beef patty with no organ inclusion; heart 6% = ground beef patty formulated with 6% beef heart; heart 12% = ground beef patty formulated with 12% beef heart; kidney 6% = ground beef patty formulated with 6% beef kidney; kidney 12% = ground beef patty formulated with 12% beef kidney. Prices used for estimation: $13.25/kg for 80:20 beef trimmings; $8.27/kg for beef heart; and $4.96/kg for beef kidney.

## Data Availability

The original contributions presented in this study are included in this article; further inquiries can be directed to the corresponding author.

## Abbreviations

The following abbreviations are used in this manuscript:

IRB	Institutional Review Board
AKSF	Allo–Kramer Shear Force
SEM	Standard error of the mean

## References

[1] Tornberg E. (2005). Effects of heat on meat proteins-Implications on structure and quality of meat products. Meat Sci..

[2] Hughes J.M., Oiseth S.K., Purslow P.P., Warner R.D. (2014). A structural approach to understanding the interactions between color, water-holding capacity and tenderness. Meat Sci..

[3] Warner R.D. (2017). The eating quality of meat—IV Water-holding capacity and juiciness. Meat Sci..

[4] Carpenter C.E., Cornforth D.P., Whittier D. (2001). Consumer preferences for beef color and packaging did not affect eating satisfaction. Meat Sci..

[5] Jeremiah L.E., Dugan M.E.R., Aalhus J.L., Gibson L.L. (2003). Assessment of the chemical and cooking properties of the major beef muscles and muscle groups. Meat Sci..

[6] Beal T., Ortenzi F. (2022). Priority micronutrient density in foods. Front. Nutr..

[7] Fuerniss H.F., Gifford C.L., Mortensen E.G., Belk K.E., Engle T.E., Woerner D.R. (2024). Nutrient analysis of raw United States beef offal items. Nutrients.

[8] Latoch A., Stasiak D.M., Siczek P. (2024). Edible offal as a valuable source of nutrients in the diet—A review. Nutrients.

[9] Valenzuela C., de Romaña D.L., Olivares M., Morales M.S., Pizarro F. (2009). Total iron and heme iron content and their distribution in beef meat and viscera. Biol. Trace Elem. Res..

[10] Pereira P.M.C.C., Vicente A.F.R.B. (2013). Meat nutritional composition and nutritive role in the human diet. Meat Sci..

[11] Devatkal S.K., Mendiratta S.K., Kondaiah N. (2004). Quality characteristics of loaves from buffalo meat, liver and vegetables. Meat Sci..

[12] Toldrá F., Reig M., Mora L. (2021). Management of meat by- and co-products for improved processing sustainability. Meat Sci..

[13] Leroy F., Cofnas N. (2020). Should dietary guidelines recommend low red meat intake?. Crit. Rev. Food Sci. Nutr..

[14] Zhang R., Zhang H., Wang Y., Tang L.-J., Li G., Huang O.-Y., Chen S.-D., Targher G., Byrne C.D., Gu B.-B. (2022). Higher consumption of animal organ meat is associated with a lower prevalence of nonalcoholic steatohepatitis. Hepatobiliary Surg. Nutr..

[15] Volpp K.G., Berkowitz S.A., Sharma S.V., Anderson C.A.M., Brewer L.C., Elkind M.S.V., Gardner C.D., Gervis J.E., Harrington R.A., Herrero M. (2023). Food Is Medicine: A Presidential Advisory from the American Heart Association. Circulation.

[16] Douglas S.L., Bernardez-Morales G.M., Nichols B.W., Johnson G.F., Barahona-Dominguez L.S., Jessup A.P., Belk A.D., Ball J.J., Cho S., Sawyer J.T. (2024). Inclusion of beef heart in ground beef patties alters quality characteristics and consumer acceptability assessed by electronic nose and tongue technology. Foods.

[17] Ball J.J., Wyatt R.P., Lambert B.D., Smith H.R., Reyes T.M., Sawyer J.T. (2021). Influence of plant-based proteins on the fresh and cooked characteristics of ground beef patties. Foods.

[18] Purslow P.P. (2018). Contribution of collagen and connective tissue to cooked meat toughness; some paradigms reviewed. Meat Sci..

[19] Miller R.K., Papadopoulos L.S., Schilling M.W., Wheeler T.L., Gray R.A., Dikeman M.E., Claus J.R., Owens C.M., King D., Belk K.E. (2026). American Meat Science Association Research Guidelines for Cookery, Sensory Evaluation, and Instrumental Texture Measurements of Meat. Meat Muscle Biol..

[20] Ayuso P., García-Pérez P., Nieto G. (2025). New Insights and Strategies in the Nutritional Reformulation of Meat Products Toward Healthier Foods. Molecules.

[21] Latimer G.W. (2023). Official Methods of Analysis of AOAC International.

[22] King D.A., Hunt M.C., Barbut S., Claus J.R., Cornforth D.P., Joseph P., Kim Y.H., Lindahl G., Mancini R.A., Nair M.N. (2023). American Meat Science Association Guidelines for Meat Color Measurement. Meat Muscle Biol..

[23] Paredes J., Cortizo-Lacalle D., Imaz A.M., Aldazabel J., Vila M. (2022). Application of texture analysis methods for the characteriziation of cultured meat. Sci. Rep..

[24] National Institutes of Health, Office of Dietary Supplements (2022). Potassium Fact Sheet for Health Professionals. U.S. Department of Health and Human Services. https://ods.od.nih.gov/factsheets/Potassium-HealthProfessional/.

[25] Mancini R.A., Hunt M.C. (2005). Current research in meat color. Meat Sci..

[26] King D.A., Shackelford S.D., Wheeler T.L. (2011). Relative contributions of animal and muscle effects to variation in beef lean color stability. J. Anim. Sci..

[27] Van Buren J.B., Puga K.J., Hoffman K.C., Nasados J.A., Bass P.D., Colle M.J. (2023). Water binders in beef patties increase yield and extend shelf life. Transl. Anim. Sci..

[28] Sammel L.M., Claus J.R. (2003). Citric acid and sodium citrate effects on reducing pink color defect of cooked ground turkey rolls. J. Food Sci..

[29] King N.J., Whyte R. (2006). Does it look cooked? A review of factors that influence cooked meat color. J. Food Sci..

[30] Warner R., Miller R., Ha M., Wheeler T.L., Dunshea F., Li X., Vaskoska R.S., Purslow P. (2021). Meat tenderness: Underlying mechanisms, instrumental measurement, and sensory assessment. Meat Muscle Biol..

[31] Domínguez R., Pateiro M., Gagaoua M., Barba F.J., Zhang W., Lorenzo J.M. (2019). A comprehensive review on lipid oxidation in meat and meat products. Antioxidants.

[32] Banović M., Grunert K.G., Barreira M.M., Fontes M.A. (2009). Beef quality perception at the point of purchase: A study from Portugal. Food Qual. Prefer..

